# Physical restraint during inpatient treatment of adolescent anorexia nervosa: frequency, clinical correlates, and associations with outcome at five-year follow-up

**DOI:** 10.1186/s40337-020-00297-1

**Published:** 2020-06-01

**Authors:** Thomas Blikshavn, Inger Halvorsen, Øyvind Rø

**Affiliations:** 1 Regional Department for Eating Disorders, Division of Mental Health and Addiction, Oslo University Hospital, P.O. Box 4956 Nydalen, N-0424 Oslo, Norway; 2 Department of Child and Adolescent Mental Health, Akershus University Hospital, Lørenskog, Norway; 3 Division of Mental Health and Addiction, Institute of Clinical Medicine, University of Oslo, Oslo, Norway

**Keywords:** Physical restraint, Forced nasogastric tube feeding, Anorexia nervosa, Eating disorders, Adolescent

## Abstract

**Background:**

Studies of the use and effects of physical restraint in anorexia nervosa (AN) treatment are lacking. The purpose of this study was to describe the frequency of physical restraint in a specialized program for adolescents with AN, and to examine if meal-related physical restraint (forced nasogastric tube-feeding) was related to 5-year outcome.

**Method:**

Thirty-eight (66% of 58) patients with AN (mean age 15.9, SD = 1.9) admitted to a regional, specialized adolescent eating disorders (ED) inpatient unit. Patient data, including restraint episodes, were obtained from hospital records, and outcome was assessed at a 5-year follow-up.

**Results:**

A total of 201 restraint episodes occurred over 5513 days of inpatient treatment, including 109 meal-related episodes and 56 episodes to avoid self-harm. Twelve (32%) patients experienced at least one restraint episode during the admission, of which eight (21%) experienced meal-related restraint. Four patients represented 91% of all restraint episodes, experiencing 10 or more episodes during admission. Meal-related restraint was significantly associated with a higher rate of persisting ED diagnosis, but not with weight gain during admission, EDE-Q global score or BMI at follow-up.

**Conclusions:**

Restraint episodes occurred rather infrequently. A small number of patients (*n* = 4) accounted for a high proportion of episodes (91%). More knowledge is important to reduce the need for restraint in treatment for AN.

## Plain English summary

It is an important goal for health services to provide treatment voluntarily and without the use of coercion. However, in severe anorexia nervosa it is sometimes necessary to use physical restraint, that is, the patient is physically held by the staff, to provide nutrition by tube feeding. Previous studies on the use of physical restraint in anorexia nervosa are lacking, and more knowledge is important to reduce the need to use restraint in treatment.

This study describes the frequency of physical restraint in a specialized program for adolescents with anorexia nervosa, and investigates if the use of physical restraint was related to 5-year outcome. About two thirds of the patients did not experience any restraint episodes, neither to provide tube feeding, nor to avoid harm. However, a small number of patients experienced a large number of restraint episodes, i.e., four of the 38 participants (11%) accounted for 91% of all physical restraint episodes. Thus, more knowledge on patients with many restraint episodes is important to avoid escalation of resistance to treatment and further use of restraint. Patients who had experienced restraint episodes for tube feeding had a higher rate of persisting eating disorders at 5-year follow-up compared to those without restraint episodes.

## Introduction

Involuntary treatment for eating disorders (ED) is utilized as a last resort in cases where there is considerable risk to the patient that cannot be managed in a less restrictive way [1–3]. The restoration of normal body weight, which depends upon meals being administered several times daily, represents a uniquely challenging cornerstone of treatment [4]. To our knowledge, no quantitative studies until the present have examined the extent to which weight restoration is facilitated by the use of physical restraint, that is, the use of physical force to restrict or control the patient. In the literature, the general view is that interventions involving physical restraint have no inherent therapeutic effects in the treatment of children and adolescents [5] and may be associated with negative psychological outcomes [6, 7]. However, the literature has largely concerned itself with physical restraint to manage patient aggression [8, 9], and therefore may not generalize to physical restraint utilized to administer nutrition in ED.

One qualitative study specifically examined the experiences of both caregivers and patients who had previously received nasogastric tube feeding (NGT) during inpatient treatment for ED in adolescence, where more than half of the patients had been restrained due to resistance to the procedure [10]. Briefly summarized, it was found that NGT was experienced negatively at the time, but was retrospectively perceived as helpful. The majority of participants did not believe there had been any viable alternatives to NGT feeding. Neither physical resistance nor reported negative reactions to the treatment had any obvious relationship to outcome.

Studies of involuntarily-treated adolescents with anorexia nervosa (AN) suggest that some patients are subject to physical restraint for treatment purposes, but the type and frequency of restraint episodes are seldom reported. For example, a large study of all specialist inpatient ED units in Scotland between 2009 and 2011 reported that 17 of 89 adolescent patients had been formally detained, and 10 had received NGT feeding [11]. Yet the extent to which NGT was administered against the patients’ will, and/or with physical restraint was not reported. The only study to date that compared outcome for adolescent AN between involuntarily admitted patients versus those admitted with parental consent found that NGT was used in 68.8% versus 11.7% of groups, respectively, although the rate of using physical restraint to administer NGT was not mentioned [12]. A Danish register-based study of involuntary measures in the treatment of AN reported that 18% of patients had experienced at least one involuntary measure, with 2% of these patients experiencing more than 100 recorded involuntary measures [13]. Involuntary NGT feeding was the most frequent involuntary measure employed, and was most prevalent in the age group 15–17 years. However, no data were available on how many meals were delivered via NGT and/or if physical restraint had been used to administer nutrition.

A large study of restraint utilized in acute general adolescent mental health units in Norway found that a small number of patients with AN were exposed to exceptionally high levels of physical restraint [14]. Specifically, 1896 of a total of 4173 restraint episodes were related to compulsory feeding. These episodes occurred in 21 patients with AN (i.e., an average of 90 episodes per patient), from a total of 4099 registered patients. Thus, nearly half (45%) of the restraint episodes registered at Norwegian acute mental health inpatient units for adolescents were related to compulsory feeding for a small number of patients with AN.

Two recent systematic reviews on compulsory treatment in AN are supportive of a beneficial outcome of compulsory treatment [15, 16]. Only one previous study has investigated outcome in adolescents with AN that had received compulsory treatment [12], and findings also concur with the more general notion of therapeutic benefit. However, the scant research evidence on compulsory treatment in adolescents with AN may not generalize to treatment delivered via physical restraint. Research is lacking on the type, frequency and effects of use of physical restraint in treatment of AN. Thus, we do not know the extent to which physical restraint is utilized, the characteristics of patients and the treatment institutions involved, and if such events influence the course and outcome of the ED.

### Aims of the study

This study aimed to describe the use of physical restraint in a specialized inpatient ED unit for adolescents, and to investigate whether the frequency and occurrence of restraint were associated with weight gain during admission and five-year outcome. Specifically, we investigated whether patients subjected to meal-related physical restraint during the index admission would have less weight gain during the admission and poorer outcome at follow-up in terms of readmissions during the follow-up period, and/or persistence of ED diagnosis, lower BMI and higher EDE-Q global score at follow-up.

## Methods

### Setting and participants

The Regional Department for Eating Disorders (RASP) at Oslo University Hospital is a tertiary, specialized ED treatment center, with a catchment area of approximately 2.9 million people. Patients are referred from their local specialized mental health services and have received prior treatment without remission. Most adolescents have received both outpatient family-based treatment (FBT) and prior inpatient treatment. Since 2008, a family-based inpatient treatment model has been offered at RASP for adolescents, based upon the evidence for outpatient FBT as well as Norwegian policies allowing parents/guardians to accompany patients under the age of 18 years during the inpatient stay. The child and adolescent inpatient unit admits patients up to 18 years old, and has beds for a maximum of five patients and their families. Details regarding the adaptation of FBT to an inpatient treatment setting have been described elsewhere [17, 18].

The current quantitative study is part of a five-year follow-up of patients who had received family-based inpatient treatment between May 2008 and June 2014. All former patients (*n* = 58) were successfully contacted and invited to participate. No exclusion criteria were applied. Of 58 patients invited, consent was obtained from 38 (66%) of the patients. If multiple hospital admissions had occurred during the follow-up period, only data from the first admission were used. At admission, all patients had a DSM-5 diagnosis of AN. Average age at admission was 15.9 years (SD = 1.9), and mean duration of inpatient treatment was 20.3 (SD = 13.7) weeks. Four of the participants were male. When comparing the 38 participants with the 20 non-participants, we found no significant differences for the following demographic and clinical characteristics at baseline: compulsory treatment status, admission BMI percentile, duration of admission, weight gain during the admission, and age at the time of the follow-up. Furthermore, no significant differences were found regarding having an entry in the restraint protocol, or having been exposed to meal-related physical restraint.

Treatment was carried out in close collaboration with the parents with the aim to support the parents’ competency and confidence in their own ability to help their child to eat sufficiently. The ED unit emphasized avoiding the use of coercion in treatment. Coercion was only used when deemed absolutely necessary to ensure adequate nutrition and vigorous efforts to achieve patient co-operation had failed. A weekly weight gain of about one kg was recommended during the admission. The unit provided a structured regime to achieve this, i.e. by providing meal plans, prescribing supplemental nutritional drinks, or NGT feeding if the patient did not finish her/his meal, and recommending how much activity and rest the child needed. The program’s meal protocol stated that the patients had to finish every meal in 30 min, followed by another 15 min to drink supplemental nutritional drinks if required. If the meal was not completed, either with regular food or nutritional drinks, NGT feeding of the remaining nutrients was prescribed. The tube was inserted after the non-completed meal and removed following replacement of the missing nutrients, based on an assumption that the patient would manage adequate oral food-intake at the next meal. Most patients completed all their meals with regular food, or supplemental nutritional drinks, with support from their parents and nursing staff. In cases of NGT feeding, the staff strongly encouraged and emphasized efforts to enlist the patient’s collaboration. However, if the patient physically resisted prescribed NGT feeding despite these efforts, a psychiatrist or clinical psychologist with the approved competence to make decisions according to the Mental Health act could, under certain circumstances, recommend that staff use physical restraint by holding the patient to administer forced NGT feeding. Parental consent was required to administer NGT feeding for patients below the age of 16, and for those above 16 years, compulsory admission and a legally valid decision on compulsory nutrition was required. The unit did not use pharmacological restraint/sedation, or mechanical restraint.

### Procedure

Information about this study, and a consent form with a decline option, were mailed to former patients and their families. Non-respondents were contacted by telephone. Patients that agreed to participate received questionnaires by mail prior to the follow-up interview. The follow-up interview was conducted at the hospital for 27 participants, at the patients’ home (*n* = 7), by telephone (*n* = 3), and elsewhere (*n* = 1). Average age at follow-up was 20.2 years (SD = 2.6), and median time to follow-up was 5.2 years (95% CI 3.8–5.5). The interviews were conducted between May 2015 and January 2016 by five experienced clinicians. All were employed at the ED department, and four had been involved in the treatment in the unit during the study period. All interviews were recorded, and any uncertainties about diagnoses were discussed by interviewers before diagnoses were coded.

### Measures

#### Data from the treatment phase

Clinical data from the treatment phase were obtained from hospital records, including weight and height at admission and discharge. BMI percentiles for age and sex were calculated using a Norwegian version of a weight-for-height ratio calculator based on reference data from Child Growth Foundation, UK [19].

##### Data regarding physical restraint during the treatment phase

In Norway, the use of coercive measures in mental health care is regulated by the Norwegian Mental Health Act, section 4–8. The types of coercive measures regulated by the law are physical holding, mechanical restraint, seclusion and medication to avoid harm. Justifications for the use of coercive measures in section 4–8 are exclusively limited to averting serious harm to the patient, others, or property. Coercion for treatment purposes (meal-related restraint) was also recorded as a coercive measure under section 4–8. The law requires that each unit has a restraint protocol where every episode with use of restraint is entered, and that this protocol be checked by independent, official, control commissions. Data on the use of restraint for nutritional treatment were therefore available. In addition to the restraint protocol, the patient records were also examined by the first author for any described episodes of physical restraint. For each episode, it was noted on which shift in the unit it occurred, justification, duration, and number of staff involved.

#### Follow-up assessment

##### Treatment received for eating disorders during the follow-up period

Information regarding inpatient and outpatient treatment was obtained from patients during the follow-up interview. If re-admitted to our study unit, readmission data were also available from the patient records. Fourteen (39%) of the participants had received additional inpatient treatment, and 34 (89%) outpatient treatment for an ED during the follow-up period.

##### Eating disorder diagnoses at follow-up

DSM-5 ED diagnoses at follow-up were assessed by the Eating Disorder Examination 16.0 (EDE) [20]. Only the diagnostic items in the EDE were used for the present study.

##### Eating disorder symptoms

The Eating Disorder Examination Questionnaire 6.0 (EDE-Q) [21, 22] was used to measure ED symptoms during the previous 28 days. The mean of the four subscales, dietary restraint, eating concern, weight concern, and shape concern, was calculated to obtain a global EDE-Q score.

##### Body weight, height, and body mass index (BMI)

Weight and height was measured during the follow-up examinations conducted at the hospital (*n* = 17). Self-reported body weight and height from the EDE interview were used if the participant did not want to be weighed (*n* = 10) or if the interview was conducted elsewhere (*n* = 11). Body mass index (BMI; kg/m^2^) was calculated from the obtained values. Mean BMI in the group with measured weighed/height (19.9, SD = 3.3) did not differ from the group with self-reported weight/height (19.9, SD = 2.9, n.s.).

### Categorization by legal status during the treatment phase

By Norwegian law, involuntary treatment status is formally only applicable to persons at the age of consent, which is 16 years old for health-related matters. Below the age of 16, the legal basis of treatment is consent by parents/caregivers. The patients were therefore categorized into three groups based on legal status during the index admission: 1) Parental consent (< 16 years, *n* = 19), 2) Voluntary treatment (≥ 16 years, *n* = 15), and 3) Involuntary treatment (≥ 16 years, *n* = 4)).

### Statistical analyses

All analyses were done using SPSS for Windows, version 23.0. Categorical variables were analyzed using the chi-squared test, or, for expected cell numbers below 1, the Fischer’s exact test. Group comparisons for normally distributed continuous variables were made using Independent Samples T-test, and where normality could not be assumed, Mann-Whitney U-test was used. Reported *p*-values are two-tailed. Effect size estimates are Cohen’s d for normally distributed variables. For the Mann-Whitney U test, effect sizes are estimated by r, as proposed by Cohen [23], and for chi-square test results by the phi coefficient (φ). By convention, for Cohen’s d, an effect size of 0.2, 0.5, and 0.8 were considered a small, medium and large effect size, respectively, and likewise, for the effect size estimates r and φ, the numbers 0.1, 0.3, and 0.5 represent a small, medium, and large effect size [24].

The study was approved by the Regional Medical Ethics Committee.

## Results

During the study period, the only type of coercive measure utilized on the unit was physical holding (physical restraint), while other coercive measures regulated by the law, such as mechanical restraint, seclusion and medication to avoid harm, were not used.

### Number of patients subject to meal-related and non-meal related physical restraint

**Fig. 1:**
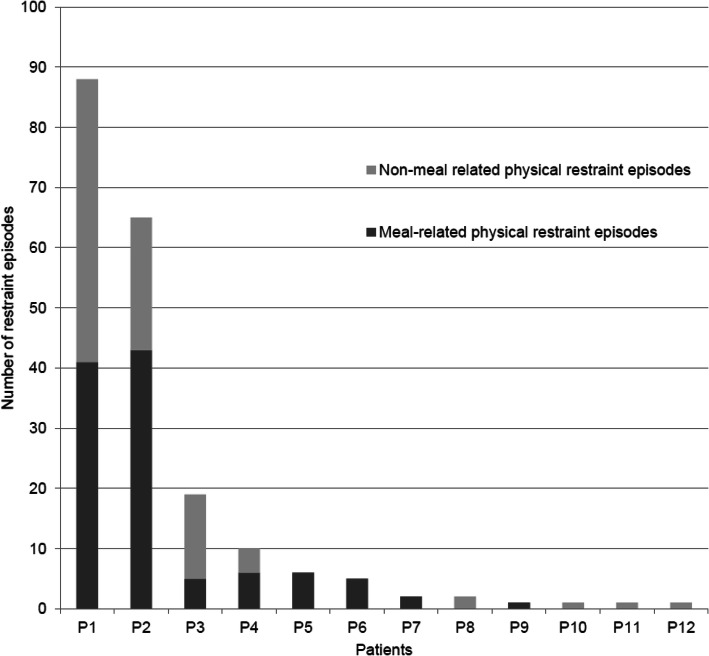
Number of physical restraint episodes among participants. Distribution of number of restraint episodes among individual patients with ≥1 restraint episode, sorted in descending order

Twelve (31%) of the 38 patients had at least one recorded episode of physical restraint, of whom eight (21%) were subject to meal-related restraint (see Fig. 1). Males and females had similar occurrence of restraint episodes. Six patients had five or more events, all of whom had at least five meal-related events, accounting for 96% of the total number of recorded restraint events. These six patients had similar clinical characteristics at admission as the other 32 patients in terms of duration of illness, age at onset, age at admission and previous inpatient treatment. Further, four of these six patients had at least 10 events, accounting for 91% of all recorded restraint events (see Fig. 1).

Among patients admitted with parental consent (*n* = 19, < 16 years), eight patients (42%) had at least one restraint episode, including five (26%) with meal-related restraint. Among the voluntarily admitted patients (*n* = 15, ≥ 16 years), there was only one single event of restraint, which was related to self-harm. Three of the four patients in the involuntary group (≥ 16 years) had physical restraint episodes, and all these three patients had more than ten restraint episodes, including both meal-related restraint and non-meal related restraint episodes. These three involuntarily admitted patients accounted for the majority (79%) of all recorded restraint episodes.

### Recorded events of physical restraint on the unit

A total of 201 occurrences of physical restraint were recorded over a total of 5513 days of inpatient treatment (treatment-days) for the 38 patients. Of the restraint episodes with a known cause (*n* = 170, see Table 1), restraint to administer nutritional treatment (54%, *n* = 109) or restraint employed to avoid serious self-harm (28%, *n* = 56) accounted together for 97% (*n* = 165) of the episodes. Across the 5513 inpatient treatment days, there was an average of 27.4 treatment-days per restraint episode for any reason, and an average of 50.6 treatment-days per meal-related restraint episode. Of the 201 recorded events, 24% of the events (*n* = 48) had missing data on at least one of the following parameters: the number of staff involved: *n* = 38 (19%), the reason for physical restraint: *n* = 31 (15%), the duration of physical restraint: *n* = 10 (5%), and the time of the day: *n* = 1. No episode of physical restraint occurred during the night shift, while 125 (63%) events occurred during day shift and 74 (37%) during the evening shift. The majority (*n* = 69, 63%) of the 109 meal-related restraint episodes took place during the first 8 weeks of the admission. However, three patients with multiple episodes during the first 8 weeks also had a large number of meal-related restraint episodes (*n* = 40) after the first 8 weeks of the admission.

**Table 1:** Numbers of physical restraint episodes in the unit, categorized by legal status of the patient and justification^a^

	Meal-related	Self-harm related	Preventing harm to others	Preventing damage to property	Unknown justification	SUM (%)
**Events during admission on parental consent (in 8 of the 19 patients on parental consent)**	19	14	3	1	4	41 (21%)
**Events during formal involuntary admission (in 3 of the 4 involuntary admitted patients)**	90	41	1	0	27	159 (79%)
	109	55	4	1	31	200 (100%)

^a^ One single event to avoid self-harm in a voluntarily admitted patient is omitted from table

### Duration of physical restraint and number of staff involved

Meal-related events (*n* = 100) had a mean duration of 19.4 min (SD = 9.2 min), while self-harm related events (*n* = 56) had a mean duration of 35.2 min (SD = 24.2 min). Of the episodes in which the number of staff involved in the event was known (*n* = 163), the mean number of staff was 3.5 (SD = 1.2) for both parental (*n* = 35) and involuntary (*n* = 127) admissions. Meal-related events (*n* = 105) had a mean number of 3.8 (SD = 1.1) staff members and self-harm related events (*n* = 53) had a mean number of 2.8 (SD = 1.0).

### Body mass index at admission and discharge

Mean BMI was 15.2 (SD = 1.9) at admission and 18.3 (SD = 1.7) at discharge, while mean BMI percentile was 5.5 (SD = 14.2) at admission and 22.9 (SD = 19.6) at discharge. Mean weight gain during the admission was 7.4 (SD = 4.5) kg. No difference was found between the group of eight patients with meal-related restraint episodes and the 30 patients without any meal-related restraint episodes regarding mean BMI and BMI-percentile at admission, discharge, or weight gain during the admission.

### Associations between meal-related physical restraint and outcome

At follow-up, sixteen (42%) of the former patients met criteria for a DSM-5 ED diagnosis (AN: *n* = 8, BN: *n* = 2, other/unspecified feeding or eating disorder: *n* = 6). The proportion with an ED diagnosis was significantly higher in the group who experienced meal-related physical restraint during admission, χ2 (1, *N* = 38) = 4.380, *p* < 0.04, φ = 0.34 (see Table 2). All the six patients with the highest numbers of restraint episodes still had an ED diagnosis at follow-up. The number of readmissions to inpatient treatment during follow-up, EDE-Q global score, and BMI at follow-up, tended to be less favorable in the group with meal-related restraint, with small-medium effect sizes, but these differences were not statistically significant.

**Table 2:** Eating disorder outcome at follow-up in patients with and without meal-related physical restraint episodes during the admission

	All patients *n* = 38	Meal-related physical restraint *n* = 8	No meal-related physical restraint *n* = 30	Test	*P*-value	Effect size^c^
Inpatient treatment during follow-up (number of patients)	14	5	9	χ^2^ (1, N = 38) = 2.792	*p* < 0.10	*φ* = 0.27
Any DSM-5 ED diagnosis at follow-up (number of patients)	16	6	10	χ^2^ (1, *N* = 38) = 4.380	*p <* 0.04	*φ* = 0.34
Follow-up EDE-Q global score (mean (SD))	2.15 (1.48)^a^	2.32 (1.52)	2.09 (1.50) ^b^	t (32) = 0.380	*p* < 0.71	*d* = 0.15
Follow-up BMI (kg/m2) (median)	19.5 (95%CI: 18.2–20.6)	18.5 (95%CI: 15.6–20.8)	19.7 (95%CI: 19.0–20.7)	U = 75.500 Z = − 1.594	*p* < 0.12	*r* = − 0.26

^a^*n* = 34, ^b^*n* = 26, ^c^by convention, for *d*, the values 0.2, 0.5 and 0.8 represent a small, medium, and large effect size, respectively, and likewise for *r* and *φ* by the values 0.1, 0.3, and 0.5

## Discussion

Approximately two-thirds of adolescent patients admitted to an inpatient family-based treatment program for AN had no recorded episode of meal-related or non-meal related physical restraint across a six-year study period. One-third of patients experienced at least one episode of either forced NGT feeding and/or physical restraint to avoid harm. Interestingly, we found that a small number of patients accounted for almost all of the restraint episodes. Specifically, six patients who had five or more restraint episodes accounted for 97% of all restraint episodes that occurred on the unit. We found that patients subjected to meal-related physical restraint had a higher rate of persistent ED diagnosis at a 5-year follow-up compared to those without a history of meal-related restraint episodes. However, there were no significant differences in the number of readmissions, BMI or EDE-Q global score at follow-up between those with versus without a meal-related restraint episode, although findings did indicate a tendency toward a more favorable outcome in patients without any meal-related restraint episodes.

To our knowledge, there are no prior studies describing the frequency of physical restraint used in the treatment of adolescent AN. Thus, it is difficult to estimate if the rate of physical restraint found in our study is lower or higher than expected. In the present study, the participants were a selected group of inpatients referred to a tertiary ED department following unsuccessful in- and outpatient treatment offered at local specialized mental health services. Their long and complex illness and treatment courses could have been associated with elevated rates of restraint episodes. Legal coercion for the treatment for AN has been found to be associated with factors such as previous admissions, complexity of the patients’ condition, and elevated health risks/low BMI [25], which are indeed factors that characterize the patient group in our study. It is likely these illness and patient-related characteristics would be associated with increased use of restraint to carry out the treatment. On the other hand, however, the treatment we provided on a specialized ED unit might have contributed to reduced use of physical restraint to administer nutrition. Staff training and expertise in meal-support therapy have been associated with greatly decreased rates of NGT feeding in this population [26], which is likely to reduce the rates of meal-related physical restraint.

The observed number of restraint episodes among the involuntary patients was particularly high, as 79% of all restraint episodes in the unit were accounted for by three of these four patients. In contrast, only one restraint episode was recorded among the 15 voluntarily admitted patients (see Table 1). A main reason for involuntary admission to treatment was that extensive past attempts to achieve weight gain voluntarily had proven unsuccessful, and legal authority to make decisions on forced nutrition was therefore deemed necessary. Forced nutrition was not legally possible to administer among voluntarily treated patients over 16 years, and, thus, there were no meal-related restraint episodes in this group. A larger proportion of patients below 16 years had at least one meal-related restraint episode (42%) compared to patients above 16 years (21%). As feeding in the younger patients was based on parental consent, forced nutrition could therefore be conducted without meeting the legal requirements and procedures necessary to establish compulsory treatment. Also, the younger, less mature patients might have poorer impulse control and more acting out, for instance in response to distress related to the treatment.

The higher number of physical restraint episodes during the day shift, and no restraint episodes during the night shift, was attributable to the unit’s procedure not to administer NGT feeding at night. Furthermore, during the process of data collection, it became evident that although the immediate behavior prevented by physical restraint was typically coded as self-harm, the behavior was often preceded by food- or meal-related events on the unit (e.g. receiving the message that calories would be increased, or having finished a meal). To the extent that food-related anxiety [27, 28] constitutes a common denominator, the relative absence of triggering food- or meal-related events at night would be relevant in explaining the observed temporal pattern.

The use of physical restraint in the treatment of AN involves complex ethical dilemmas for health authorities, institutions, as well as the staff that carries out the restraint. Physical restraint might also be extremely resource-intensive and demanding, both financially and with respect to the staff’s expertise. We found that each restraint episode involved a large number of staff (mean 3.5, SD = 1.2) for a significant time period (mean 22.9 min, SD = 16.9). The meal-related restraint episodes were particularly demanding given the challenges involved in administering safe tube-feeding while patients physically resisted the procedure. Typically, most of the staff on the unit were involved in the restraint episodes to prevent harm, which could not be planned beforehand, and staff was often “borrowed” from another unit to carry out meal-related restraint. During admissions of patients with numerous physical restraint episodes, extra staff had to be engaged. Restraint episodes were also demanding for the specialists responsible for treatment, as each episode required assessment, decision-making, and documentation by a psychiatrist or clinical psychologist certified with the approved competence to make decisions based on the Mental Health Act. Additionally, the use of physical restraint, particularly to administer forced feeding, is demanding and distressing for the staff, the patient, her/his family, as well as other patients and families admitted to the unit. Thus, achieving the successful re-nourishment in AN inpatient treatment which minimizes the use of physical restraint, is important for ethical reasons, health service economy, staff resources and well-being, as well as for the adolescents affected by AN and their families.

The highly skewed distribution of physical restraint episodes across patients is in concert with prior research, including a study on the use of involuntary measures in AN [13], as well as studies from outside the ED field, such as general adolescent inpatient populations in Norway [29], single adolescent psychiatric facilities in the US [30, 31], and child and adolescent inpatient treatment settings [32, 33]. Moreover, similar findings are reported for adult patients [34–36].

Our findings entail several important implications for the use of physical restraint and management during inpatient treatment in adolescent AN. First, interventions delivered ubiquitously to all patients in order to reduce the probability of physical restraint may not be very effective in reducing overall numbers, as the majority of patients experience little or no physical restraint. Additional investigation of those with frequent physical restraint episodes may lead to a better understanding of how to reduce or eliminate restraint in this group. Second, and in line with the health authorities’ focus on reduced use of involuntary treatment and coercive measures in mental health services, treatment units should aim to deliver treatment voluntarily as much as possible. However, the reduction of restraint episodes might be obtained by selective targeting, with treatment units achieving substantial reductions in restraint episodes by subtle changes in admission policy, transfer of selected patients to another unit, or early discharge, none of which would be of any apparent benefit to the patients in question [36]. Because of this, further studies on physical restraint in the treatment of EDs should ideally include multiple sites, and also account for the amount of physical restraint delivered in non-psychiatric settings, particularly in somatic units. Finally, countertransference reactions appear important in understanding the clinician-patient dynamics in adolescent AN [37]. Also, staff emotional reactions appear relevant in understanding the escalation and persistence of patient aggression [38], which might be relevant for restraint rates. In our opinion, the contribution of clinician emotional reactions and behaviors to the emergence of coercion in treatment settings in general, and particularly to escalation of forced NGT feeding episodes, deserves further investigation.

Emotion regulation difficulties may also contribute to the emergence of physical restraint in the treatment setting, but the present study is not able to elucidate this question. Emotion regulation difficulties may be unrelated to weight status, and appear to be insensitive to weight normalization, at least in the short term [39]. This may particularly inform our understanding of patients with recurrent physical restraint episodes. Qualitative data suggest that awareness and tolerance of negative emotions, both of which are central tasks in emotion regulation, are central to the recovery process, or, conversely, that lack thereof contribute to relapse after weight restoration [40]. It could be of clinical relevance to include measures related to emotion regulation difficulties in future research on physical restraint in AN treatment.

In the present study, patients with meal-related restraint did not differ significantly from those with no meal-related restraint in terms of clinical characteristics or weight status during admission. Further, differences in the number of readmissions and BMI and EDE-Q global score at follow-up did not reach statistical significance. However, the proportion of patients who had an ED diagnosis at 5-year follow-up was significantly higher in the group who experienced meal-related coercion during hospitalization, with a medium effect size (φ = 0.34). Given the small number of patients exposed to meal-related restraint, however, both negative and positive findings should be interpreted with caution. Notably, all outcome measures at follow-up trended toward a poorer outcome for those who experienced meal-related coercion during admission, and, except for the EDE-Q global score, effect sizes were consistent with a medium effect. Also, it is noteworthy that all the six patients with highest numbers of restraint episodes still had an ED diagnosis at follow-up. In sum, we believe that the current study, being the first of its kind, suggests that a history of meal-related restraint may be of clinical importance to long-term outcome, justifying further investigation of this clinically challenging problem.

The main limitation of the current study is the small number of patients in the group of interest, leading to a high risk of type II errors. About 1/3 of those treated during the study period did not participate in this follow-up study, although the non-participants did not significantly differ from participants on a number of background variables, including formal involuntary status, registered restrained episodes, or the presence of meal-related physical restraint, which lends support to the representativeness of the results for our patient group. The focus of the study was on physical restraint, but, as the few involuntary patients accounted for the majority (79%) of the restraint episodes, we are not able to distinguish the effect of having experienced physical restraint episodes on outcome from the effect of having received involuntary treatment. Also, as the study population is from a tertiary treatment center, the participants are a highly selected group, and may not be representative of other inpatients settings for ED. Legislation and organization of health services for the most severely ill patients with AN differ between countries, which also constitutes a limitation in the generalizability of the results of this study.

## Conclusion

The use of involuntary treatment and coercive measures in mental health services entails complex ethical dilemmas, especially for the treatment of AN, in which life and physical health might be at risk and patients often refuse medically necessary procedures to ensure weight gain. More knowledge of the use of physical restraint in the treatment of adolescent AN is important, and, if possible, a reduction in the use of such measures. On this tertiary inpatient unit for adolescents with severe AN, the occurrence of physical restraint was rather infrequent. Only a small number of patients accounted for the vast majority of physical restraint episodes. Physical restraint seemed to be related to poorer prognosis at a 5-year follow-up, as evidenced by the persistence of an ED diagnosis. Additional research with larger samples is necessary to better understand the use and effects of physical restraint in AN.

## Data Availability

The dataset analyzed during the current study is kept on a secure research server at Oslo University Hospital. It is not publicly available due to data protection regulations for medical research. Please contact the first author if information is requested.

## Abbreviations

ED: Eating disorder
NGT: Nasogastric tube feeding
AN: Anorexia nervosa
BMI: Body mass index (kg/m^2^)
FBT: Family-based treatment
EDE-Q: The eating disorder examination questionnaire
